# Dietary and Environmental Exposure to Toxic Elements in Endometriosis: A Missing Piece of the Etiological Puzzle?

**DOI:** 10.3390/nu18152489

**Published:** 2026-08-02

**Authors:** Kamila Pokorska-Niewiada, Izabela Gutowska, Małgorzata Szczuko

**Affiliations:** 1 Department of Toxicology, Dairy Technology and Food Storage, West Pomeranian University of Technology, 71-454 Szczecin, Poland; 2 Department of Medical Chemistry, Pomeranian Medical University, 70-204 Szczecin, Poland; izabela.gutowska@pum.edu.pl; 3 Department of Bromatology and Diagnostic Nutrition, Pomeranian Medical University, 70-111 Szczecin, Poland

**Keywords:** endometriosis, cadmium, toxic elements, biomonitoring, environmental exposure, nutritional status, iron homeostasis, endocrine disruption

## Abstract

**Background/Objectives:** Endometriosis is a chronic estrogen-dependent disease with a complex and incompletely understood etiology. Environmental exposure to toxic elements, including cadmium, lead, mercury, and arsenic, may contribute to its development and progression. Proposed mechanisms include endocrine disruption, oxidative stress, chronic inflammation, and epigenetic alterations. **Methods**: This narrative review was based on a structured literature search of the PubMed, Scopus, and Web of Science databases for studies published up to 15 June 2026. The search included the following keywords: endometriosis, cadmium, lead, mercury, arsenic, toxic elements, heavy metals, environmental exposure, diet, nutrition, and biomonitoring. **Results:** This review summarizes current evidence on the relationship between toxic element exposure and endometriosis, including sources of exposure, biomonitoring findings, proposed biological mechanisms, and human studies. The influence of nutritional status on metal bioavailability is also considered. Among the toxic elements reviewed, cadmium has been studied most extensively and shows the most consistent association with endometriosis. Evidence for lead is less consistent, whereas data on mercury and arsenic remain limited. Although the available epidemiological evidence does not establish a causal relationship, biomonitoring and experimental studies support a possible role of toxic elements in the development and progression of endometriosis. **Conclusions:** Current evidence supports a possible role of toxic elements in endometriosis, although a causal relationship has not been established. Cadmium has been investigated most extensively, while evidence for other toxic elements remains limited. Nutritional status should be considered when evaluating biomonitoring findings, particularly in relation to cadmium exposure.

## 1. Introduction

Chronic exposure to toxic elements is an unavoidable part of modern life. In the general population, diet is the primary route of exposure, and cadmium (Cd), lead (Pb), mercury (Hg), and arsenic (As) are among the best-studied environmental contaminants with documented effects on human health. This review focuses on these four elements because they are among the most common toxic elements in environmental exposure and have been studied extensively in relation to endocrine and reproductive toxicity. Their persistence, long biological half-lives, bioaccumulation, and ability to disrupt endocrine and immune function mean that even chronic exposure to low doses may have biological consequences [1,2,3]. One area in which the role of toxic elements remains poorly understood is endometriosis. This chronic estrogen-dependent disease affects approximately 10% of women of reproductive age and is one of the leading causes of chronic pelvic pain and infertility. Although retrograde menstruation remains the most widely accepted theory of disease development, it does not explain why only a subset of women develop endometriosis, suggesting that additional genetic, immunological, hormonal, and environmental factors are involved [4,5].

Toxic elements are of particular interest because they may interfere with several biological processes implicated in endometriosis, including estrogen signaling, chronic inflammation, oxidative stress, and epigenetic regulation. Cadmium has attracted particular attention because of its metalloestrogenic activity and its ability to mimic the effects of estrogens [6].

An increasing number of epidemiological and biomonitoring studies have investigated the association between toxic element exposure and endometriosis. Toxic elements have been measured not only in blood and urine but also in follicular fluid, endometrial and ovarian tissues, and peritoneal fluid. However, the available findings remain inconsistent, probably owing to differences in study design, exposure assessment, biological matrices, and diagnostic criteria [7,8,9,10,11,12,13,14,15]. From a nutritional perspective, the interaction between iron status and cadmium toxicokinetics appears particularly relevant. Iron deficiency, which is common among women with endometriosis, may increase intestinal cadmium absorption through shared metal transport pathways. This suggests that internal cadmium burden depends not only on environmental exposure but also on nutritional status [16,17].

The aim of this review is to critically evaluate current evidence on the association between toxic element exposure and endometriosis, with particular emphasis on sources of exposure, biomonitoring, biological mechanisms, and the potential role of nutritional status as a modifier of internal metal exposure.

## 2. Materials and Methods

This study was conducted as a narrative review based on a structured search of the scientific literature. Searches were performed in the PubMed, Scopus, and Web of Science databases to identify publications available up to 15 June 2026. The search was performed using combinations of the keywords “endometriosis” with “cadmium”, “lead”, “mercury”, “arsenic”, “toxic elements”, “heavy metals”, “environmental exposure”, “diet”, “nutrition”, and “biomonitoring”. Different keyword combinations were used in each database according to its search interface. Separate searches were performed for each toxic element. The reference lists of relevant articles were also screened to identify additional publications that were not retrieved during the initial database search.

Original epidemiological studies, biomonitoring studies, experimental studies, and relevant review articles published in English were considered for inclusion. Studies focusing on cadmium, lead, mercury, or arsenic in relation to endometriosis or biological mechanisms potentially involved in its pathogenesis were included. When more than one publication reported results from the same study population, the most comprehensive or recent report was considered. Conference abstracts, editorials, letters to the editor, duplicate publications, and case reports were excluded.

The retrieved records were screened by title and abstract, followed by full-text evaluation of potentially relevant articles. Publications were assessed for relevance based on the predefined inclusion and exclusion criteria. The search identified 1416 records. After duplicate removal and eligibility assessment, relevant publications were selected for inclusion in this narrative review. The final reference list comprised 106 publications, including nine original human studies evaluating the association between toxic element exposure and endometriosis, which are summarized in table.

The included studies were evaluated with respect to exposure sources, biomonitoring findings, biological mechanisms, epidemiological evidence, and the potential influence of nutritional status on toxic element absorption and internal exposure.

## 3. Dietary and Environmental Exposure to Toxic Elements

Exposure to toxic elements is widespread and affects virtually the entire population. Unlike many other environmental contaminants, exposure is not limited to specific occupational groups or geographic regions.

For most individuals, the primary sources of cadmium, lead, mercury and arsenic are everyday dietary intake, while environmental pollution, tobacco smoking and occupational exposure further contribute to the overall body burden [18,19,20,21,22]. Owing to their ability to bioaccumulate, even low-level exposure over many years may lead to the accumulation of biologically relevant concentrations in tissues (Table 1).

### 3.1. Diet as the Main Route of Chronic Exposure

**Figure 1:**
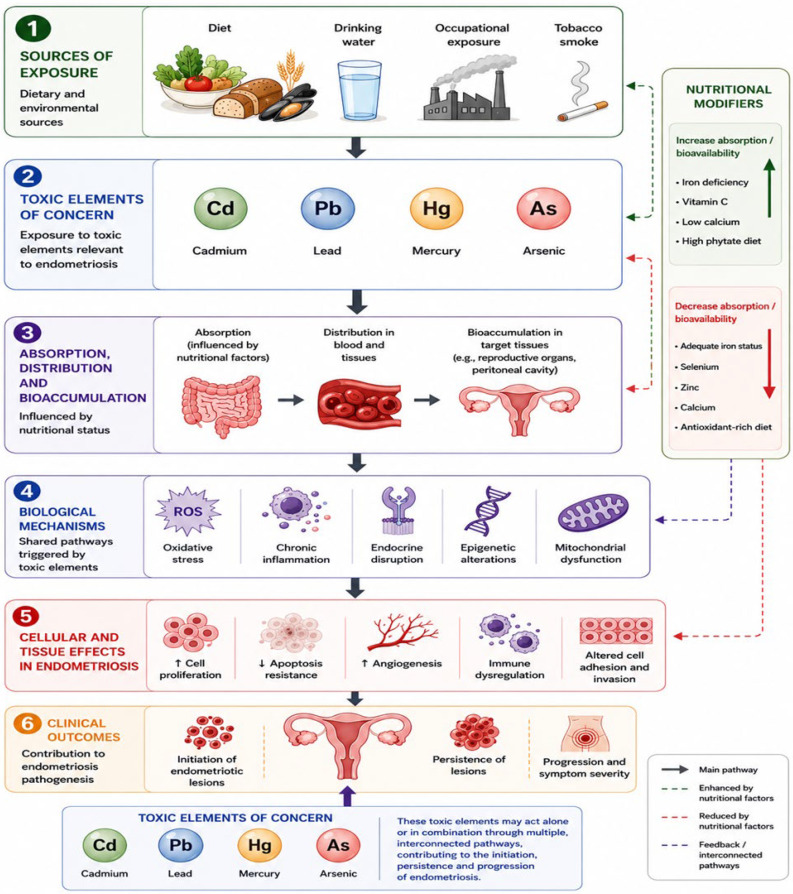
Integrated framework of toxic element exposure, nutritional modifiers and biological mechanisms in endometriosis.

Diet is the primary source of chronic exposure to toxic elements in the general population. Their concentrations in food depend on multiple factors, including contamination of soil and water, agricultural practices, geographic location, and food processing methods [18,20,23,31,38]. Cereals, rice, potatoes, and leafy vegetables are the main dietary sources of cadmium. Arsenic exposure occurs primarily through drinking water and rice-based products, whereas mercury exposure is mainly associated with the consumption of fish and seafood, particularly predatory species (Table 1, Figure 1). Lead may be present in both food and drinking water, especially in areas affected by industrial pollution. It is noteworthy that some foods considered part of a healthy diet may also contribute to long-term exposure to toxic elements. This highlights the importance of food quality and long-term exposure assessment, particularly in populations at increased risk [18,19,20,21,22,39,40].

### 3.2. Environmental and Occupational Exposure

In addition to diet, environmental contamination remains an important source of exposure. Industrial emissions, fossil fuel combustion, mining activities, and contamination of soil and surface waters contribute to the release of toxic elements into the food chain [41,42,43,44]. In some regions of the world, elevated arsenic concentrations in groundwater remain a major public health concern [37,45]. In addition to dietary and environmental exposure, tobacco smoking is an important source of cadmium exposure. Blood and urinary cadmium concentrations are generally higher in smokers than in non-smokers [33,46]. Additional exposure occurs in certain occupational settings, particularly in the metallurgical, electronics, chemical and mining industries [47].

### 3.3. Bioaccumulation and Factors Modifying Internal Exposure

The health significance of toxic elements is determined not only by the widespread nature of exposure but also by their toxicokinetic properties. Cadmium, lead and mercury have long biological half-lives and accumulate gradually in the body. As a result, tissue concentrations reflect long-term cumulative exposure rather than recent dietary intake or short-term environmental exposure [21,24,48].

Internal exposure is determined not only by the level of environmental exposure but also by nutritional status. One of the best-characterized examples is the relationship between iron homeostasis and cadmium absorption. Iron and cadmium share common intestinal transport pathways, including the divalent metal transporter 1 (DMT1). Consequently, iron deficiency enhances cadmium absorption and promotes its accumulation in the body [49,50].

This interaction may be particularly relevant in women with endometriosis. Heavy menstrual bleeding and chronic blood loss frequently lead to depleted iron stores, which may further increase intestinal cadmium absorption. Therefore, nutritional status may not only reflect disease burden but also influence individual susceptibility to the adverse effects of toxic elements [51,52]. This concept may partly explain why individuals with comparable environmental exposure differ in their internal body burden of cadmium and, potentially, in their biological response to exposure. The key information on exposure sources, toxicokinetic and potential mechanisms of action of the analyzed toxic elements is summarized in Table 1 and Figure 1.

## 4. Biomonitoring of Toxic Elements in Women with Endometriosis

For many years, the assessment of exposure to toxic elements has relied primarily on measurements of their concentrations in blood and urine [10,53,54]. Although these biomarkers provide information on systemic exposure, they do not indicate whether toxic elements accumulate in tissues of the female reproductive tract [5,12]. Consequently, recent studies have increasingly focused on biological samples collected directly from reproductive tissues, including the endometrium, ovarian tissue, follicular fluid and peritoneal fluid. These analyses provide insight into the local accumulation of toxic elements and their potential influence on the local microenvironment that supports the establishment and persistence of endometriotic lesions [5,14,55,56].

### 4.1. Endometrial and Ovarian Tissues

Toxic elements have been detected in both the endometrium and ovarian tissue. Elemental analyses of endometrial tissue have demonstrated differences between healthy and diseased samples, suggesting disturbances in local elemental homeostasis. Cadmium and lead have also been detected in ovarian tissue, with cadmium concentrations increasing with age [55].

Additional evidence comes from studies involving women with infertility. Higher cadmium concentrations have been reported in the endometrium of women with unexplained infertility than in fertile controls [11,57,58]. These findings support the possibility that cadmium accumulates in endometrial tissue, where it may influence the local environment required for embryo implantation. Although these studies were not limited to women with endometriosis, they support the concept that toxic elements can accumulate in the female reproductive tract and potentially influence reproductive function.

### 4.2. Follicular and Peritoneal Fluid

Follicular fluid provides valuable information about the local environment in which oocyte maturation occurs. Studies involving women with endometriosis have detected cadmium, lead, mercury and arsenic in both blood and follicular fluid, with higher concentrations observed than in control groups. For lead, higher follicular fluid concentrations have also been associated with an increased risk of endometriosis [9,59].

Far fewer studies have examined peritoneal fluid. However, the available evidence suggests elevated concentrations of selected metals, including lead and nickel, in the immediate vicinity of endometriotic lesions [60,61]. Although these findings are based on a limited number of studies, they support the possibility of local accumulation of toxic elements within the pelvic cavity.

### 4.3. Clinical Significance of Tissue Biomonitoring

Studies of reproductive tissues and biological fluids complement the information obtained from blood and urine biomonitoring. They demonstrate that toxic elements are not confined to the systemic circulation but can also accumulate in tissues and fluids directly involved in reproductive function and the development of endometriosis.

However, it remains unclear whether local concentrations primarily reflect long-term environmental exposure, altered tissue metabolism, or both. Likewise, their potential value as biomarkers of disease presence or progression has not yet been established. Future studies integrating systemic and tissue-specific biomonitoring may improve our understanding of the biological relevance of local toxic element accumulation in endometriosis.

Collectively, these findings indicate that biomonitoring of reproductive tissues provides information that cannot be obtained from blood or urine alone and may improve our understanding of the role of toxic elements in endometriosis.

## 5. Biological Mechanisms Linking Toxic Elements and Endometriosis

The association between toxic element exposure and endometriosis has not yet been conclusively established. However, experimental studies provide evidence for several biologically plausible mechanisms through which cadmium, lead, mercury and arsenic may contribute to disease development. These elements affect multiple biological pathways, including endocrine signaling, inflammatory responses, oxidative balance and gene regulation, all of which are closely involved in the pathogenesis of endometriosis [13,17,62].

### 5.1. Endocrine Disruption

Endometriosis is an estrogen-dependent disease, and disturbances in hormonal regulation are considered a key component of its pathogenesis. Increased local estrogen production together with reduced progesterone responsiveness promotes the proliferation, survival and persistence of ectopic endometrial lesions [63].

Among toxic elements, cadmium has the best-characterized endocrine activity and is classified as a metalloestrogen because of its ability to interact with estrogen receptors. Experimental studies have shown that cadmium can activate estrogen-dependent signaling pathways and increase aromatase activity, thereby enhancing local estrogen synthesis [64,65]. These effects are consistent with the hormonal alterations observed in endometriosis and may contribute to the establishment and progression of endometriotic lesions.

Experimental evidence also suggests that toxic elements may alter the expression of estrogen receptors and impair progesterone signaling. Although these observations are based mainly on in vitro and animal studies, they support a biologically plausible link between environmental exposure and endocrine pathways involved in endometriosis [7,66,67].

Much less is known about the endocrine effects of lead, mercury and arsenic. Available evidence suggests that these elements may also interfere with hormonal signaling; however, their contribution to the pathogenesis of endometriosis remains uncertain.

### 5.2. Oxidative Stress and Inflammation

Chronic inflammation and oxidative stress are among the key mechanisms involved in the pathogenesis of endometriosis. Endometriotic lesions are characterized by increased production of reactive oxygen species (ROS), activation of immune cells and elevated levels of pro-inflammatory cytokines. Together, these processes promote cell proliferation, angiogenesis and the persistence of ectopic lesions [68]. Toxic elements may exacerbate these processes by increasing ROS production and reducing the activity of endogenous antioxidant enzymes, including superoxide dismutase (SOD), catalase (CAT) and glutathione peroxidase (GPx). As a consequence, oxidative damage to lipids, proteins and DNA promotes the activation of inflammatory signaling pathways [69,70,71,72].

The relevance of these mechanisms has also been demonstrated in studies using human endometrial stromal cells. Palomar et al. [55] reported that mercury exposure increased ROS production, impaired decidualization, reduced prolactin and IGFBP-1 secretion, and promoted apoptosis. These findings suggest that mercury may directly impair endometrial function through oxidative stress–related mechanisms. Another important component of the oxidative stress response is activation of the transcription factor NF-κB, which regulates the expression of numerous inflammatory mediators, including TNF-α, IL-1β and IL-6 [73,74]. Persistent activation of these pathways may promote the survival of ectopic endometrial cells, angiogenesis and tissue remodeling, all of which are hallmarks of endometriosis. Although most evidence comes from experimental studies, the observed mechanisms are consistent with pathological processes identified in women with endometriosis. Together, these findings support oxidative stress and chronic inflammation as biologically plausible links between toxic element exposure and the development of endometriosis.

### 5.3. Iron Metabolism and Toxic Element Absorption

Disturbances in iron homeostasis are a common feature of endometriosis and may influence not only the clinical course of the disease but also internal exposure to toxic elements. Heavy menstrual bleeding and chronic blood loss, frequently observed in women with endometriosis, may lead to depleted iron stores and iron deficiency [51]. Iron deficiency increases the expression of divalent metal transporter 1 (DMT1) in the intestinal epithelium. Because DMT1 transports both iron and cadmium, increased transporter activity may enhance intestinal cadmium absorption [75,76]. As a result, women with low iron stores may accumulate higher amounts of cadmium despite similar levels of environmental exposure. This mechanism may partly explain the interindividual differences in cadmium concentrations reported in biomonitoring studies. More importantly, it highlights that the internal body burden of toxic elements is determined not only by environmental exposure but also by nutritional status [17,76]. The interaction between iron deficiency and cadmium absorption highlights that internal cadmium burden depends not only on environmental exposure but also on individual iron status. This mechanism may partly explain the variability in cadmium concentrations observed among women with similar levels of environmental exposure.

### 5.4. Epigenetic Mechanisms

Epigenetic alterations are recognized as an important component of endometriosis pathogenesis. Abnormal DNA methylation, histone modifications and altered microRNA expression have been reported in endometrial tissues and may contribute to dysregulated cell proliferation, inflammation, angiogenesis and hormonal signaling [77]. Experimental studies indicate that toxic elements can induce similar epigenetic changes. Exposure to cadmium, arsenic, lead and mercury has been shown to affect DNA methylation, chromatin structure and microRNA expression, leading to persistent changes in gene regulation [78,79,80]. These mechanisms may represent one of the pathways linking long-term environmental exposure to the development of endometriosis.

However, direct evidence supporting the involvement of these epigenetic mechanisms in women with endometriosis remains limited. Most available data come from experimental studies, highlighting the need for well-designed clinical investigations to determine the relevance of these epigenetic changes in women with endometriosis [17,81,82].

Collectively, these mechanisms provide biological plausibility for the hypothesis that toxic elements may contribute to the development and progression of endometriosis. The proposed mechanisms are likely to be interconnected rather than acting independently. For example, oxidative stress may enhance inflammatory responses, whereas chronic inflammation may contribute to epigenetic alterations involved in lesion persistence. Endocrine disruption may further modify these pathways through altered hormonal signaling. However, most available evidence comes from experimental studies, and confirmation in women with endometriosis remains limited.

## 6. Human Evidence Linking Toxic Elements and Endometriosis

Published studies investigating the association between toxic element exposure and endometriosis differ considerably in study design, exposure assessment, biomarkers, and diagnostic criteria. The studied populations included women from the general population, patients undergoing laparoscopy, and women evaluated or treated for infertility. Toxic element concentrations were measured in a variety of biological matrices, including blood, urine, follicular fluid and reproductive tissues [83,84]. This methodological heterogeneity makes direct comparison of study findings difficult and is likely one of the main reasons for the inconsistent results reported to date. The main characteristics and findings of the available human studies are summarized in Table 2.

### 6.1. Cadmium

Cadmium is the most extensively studied toxic element in relation to endometriosis, although the available findings remain inconsistent. Some studies have reported a positive association between cadmium exposure and endometriosis, whereas others have found no significant relationship.

One of the first studies to suggest such a link reported a dose–response relationship between blood cadmium concentrations and the risk of endometriosis [75]. In contrast, Itoh et al. [85] and Heilier et al. [86] did not observe significant differences in cadmium concentrations between women with endometriosis and healthy controls.

More recent studies have provided additional evidence for a possible association between cadmium exposure and endometriosis. In an analysis of NHANES data, Hall et al. [8] found a positive association between urinary cadmium concentrations and the prevalence of endometriosis. Similarly, Shen et al. [15] reported higher cadmium concentrations in both blood and follicular fluid of women with endometriosis.

The inconsistent findings across studies may reflect differences in exposure biomarkers, diagnostic criteria and study populations. Despite these limitations, cadmium remains the toxic element with the strongest body of evidence supporting a potential association with endometriosis.

### 6.2. Lead

Compared with cadmium, fewer studies have evaluated the association between lead exposure and endometriosis. Nevertheless, the available evidence suggests that lead may also be involved in disease development. Some studies have reported higher lead concentrations or positive associations with endometriosis, whereas others found no significant association [15,25].

Additional evidence comes from studies of occupational exposure. Kim et al. [87] reported an increased risk of hospitalization for endometriosis among women exposed to lead and suggested a possible combined effect of lead and cadmium. However, these findings have not been consistently confirmed, and the overall evidence remains limited.

### 6.3. Mercury

Much less is known about the role of mercury in endometriosis. Epidemiological evidence is limited and is based mainly on a small number of biomonitoring studies. While Shen et al. [15] reported higher mercury concentrations in both blood and follicular fluid of women with endometriosis, other epidemiological studies found no significant association between mercury exposure and endometriosis [13,75,86].

Most evidence regarding mercury comes from experimental studies. Mercury exposure has been shown to impair the function of human endometrial stromal cells, increase oxidative stress and disrupt decidualization, suggesting a potential adverse effect on endometrial function [2,55,84]. Nevertheless, the available clinical evidence remains insufficient to define the role of mercury in the development of endometriosis.

**Table 2:** Characteristics and main findings of epidemiological studies evaluating cadmium, lead, mercury, and arsenic exposure in relation to endometriosis.

Study	Country	Study Design	Endometriosis Diagnosis	Population/Sample Size	Biological Matrix	Analytical Method	Main Findings	Effect Estimate	Adjusted Covariates	Main Limitations
Cadmium (Cd)										
[75]	USA	cross- sectional (NHANES)	self-reported	women 20–49 years (*n* = 1425; 61 cases)	blood	AAS	higher Cd associated with increased endometriosis odds	T2 vs. T1: aOR 1.94 (95% CI 0.73–5.18); T3 vs. T1: aOR 3.39 (95% CI 1.37–8.40)	age, race/ethnicity, smoking, OC use	cross-sectional; self-reported diagnosis
[85]	Japan	hospital case–control	laparoscopically confirmed	54 cases; 74 controls	urine	ICP-MS	no significant association	aOR 0.86 (0.30–2.49)	menstrual cycle length, BMI, smoking	small sample; infertile women only
[86]	Belgium	hospital case–control	surgically confirmed	119 cases; 25 controls	blood, urine	GF-AAS	no difference in Cd concentrations	concentration comparison only	age, smoking	small control group
[13]	USA	matched cohort (ENDO)	surgery/MRI	operative: *n* = 473; population *n* = 131	blood, urine	ICP-MS	no overall association; inverse association in the operative cohort	blood Cd (operative): aOR 0.55 (0.31–0.98)	age, BMI, smoking, site, race, vitamin use, urinary creatinine	cross-sectional; heterogeneous diagnosis
[8]	USA	cross- sectional (NHANES)	self-reported	women 20–54 years (*n* = 1647; 108 cases)	urine	ICP-MS	higher Cd levels associated with increased endometriosis odds	Q2 vs. Q1: aPR 2.0 (1.1–3.9); Q3 vs. Q1: aPR 2.0 (1.1–3.7)	age, smoking, education, urinary creatinine	cross-sectional design; self-reported diagnosis
[15]	China	hospital case–control	surgically confirmed	609 women; (blood: 234/217; follicular fluid: 203/182)	blood, follicular fluid	ICP-MS	higher Cd levels associated with increased endometriosis odds	blood: T2 aOR 1.96 (1.07–3.58); T3 aOR 3.21 (1.79–5.77). FF: T2 aOR 2.45 (1.29–4.65); T3 aOR 3.12 (1.67–5.83)	multivariable-adjusted model	hospital-based; cross-sectional; mixture effects
[58] *	Türkiye	case–control	not applicable	33 unexplained infertility; 32 fertile controls	endometrial tissue	AAS	higher endometrial Cd in unexplained infertility	aOR 18.9 (4.5–79.9)	age; BMI	indirect evidence
Lead (Pb)										
[86]	Belgium	hospital case–control	surgically confirmed	119 cases; 25 controls	blood, urine	GF-AAS	no significant difference in Pb concentrations	concentration comparison only	age; smoking	small control group
[13]	USA	matched cohort (ENDO)	surgery/MRI	operative: *n* = 473; population: *n* = 131	blood, urine	ICP-MS	no association	blood Pb aOR = 0.84 (95% CI 0.50–1.41)	age, BMI, smoking, site, race, vitamin use, urinary creatinine	cross-sectional; heterogeneous diagnosis
[15]	China	hospital case–control	surgically confirmed	609 women; (blood: 234/217; follicular fluid: 203/182)	blood, follicular fluid	ICP-MS	higher Pb levels associated with increased endometriosis odds	blood T3 vs. T1: aOR 2.73 (95% CI 1.56–4.78); follicular fluid T2 vs. T1: aOR 1.97 (95% CI 1.11–3.52)	multivariable adjusted model	hospital-based; cross-sectional
[88]	Italy	prospective score-matched case–control	histologically confirmed	13 cases; 33 infertile controls	follicular fluid	GF-AAS	higher Pb levels associated with increased endometriosis odds	OR 14.6 (95% CI 2.50–142.59)	none, propensity score matching	small exploratory cohort; infertile women only
[87] **	South Korea	occupational cohort	hospital records	26,542 women	blood	occupational biomonitoring	occupational Pb/Cd co-exposure associated with increased endometriosis odds	interaction analysis (RERI = 0.33)	occupational exposure variables	occupational setting
Mercury (Hg)										
[75]	USA	cross- sectional (NHANES)	self-reported	women 20–49 years (*n* = 1425; 61 cases)	blood	ICP-MS	no association	no significant association	age, race/ethnicity, smoking, OC use	cross-sectional design; self-reported diagnosis
[13]	USA	matched cohort (ENDO)	surgery/MRI	operative: *n* = 473; population: *n* = 131	blood, urine	ICP-MS	no association	no significant association	age, BMI, smoking, site, race, vitamin use, urinary creatinine	cross-sectional; heterogeneous diagnosis
[15]	China	hospital case–control	surgically confirmed	609 women; (blood: 234/217; follicular fluid: 203/182)	blood, follicular fluid	ICP-MS	higher Hg associated with increased endometriosis odds	blood: high vs. low aOR 13.10 (95% CI 6.74–25.44); T2 vs. T1 aOR 15.27 (95% CI 4.96–46.97); T3 vs. T1 aOR 35.66 (95% CI 11.99–106.08). Follicular fluid: T3 vs. T1 aOR 1.86 (95% CI 1.05–3.29)	multivariable- adjusted model	hospital-based; cross-sectional; mixture effects
[86]	Belgium	hospital case–control	surgically confirmed	119 cases; 25 controls	blood, urine	GF-AAS	no difference in Hg concentrations	concentration comparison only	age, smoking	small control group
Arsenic (As)										
[13]	USA	matched cohort (ENDO)	surgery/MRI	operative: *n* = 473; population: *n* = 131	urine	ICP-MS	no association	no significant association	age, BMI, smoking, site, race, vitamin use, urinary creatinine	cross-sectional; heterogeneous diagnosis
[15]	China	hospital case–control	surgically confirmed	609 women; (blood: 234/217; follicular fluid: 203/182)	blood, follicular fluid	ICP-MS	higher As associated with increased endometriosis odds	blood: highest vs. lowest tertile aOR = 5.53 (95% CI 2.97–10.30). follicular fluid: highest vs. lowest tertile aOR = 2.42 (95% CI 1.35–4.33)	multivariable-adjusted model	hospital-based; cross-sectional; mixture effects

* indirect evidence: women with unexplained infertility rather than diagnosed endometriosis; ** occupational cohort; findings may not be directly comparable with biomonitoring studies; OC—oral contraceptive; AAS—Atomic Absorption Spectrometry; GF-AAS—Graphite Furnace Atomic Absorption Spectrometry; ICP-MS—Inductively Coupled Plasma Mass Spectrometry.

### 6.4. Arsenic

Only a few studies have evaluated the association between arsenic exposure and endometriosis. Available data are limited, although one recent study reported higher arsenic concentrations in women with endometriosis. Given the widespread environmental exposure to arsenic and its well-documented effects on inflammation and epigenetic regulation, its potential role in the pathogenesis of endometriosis warrants further investigation [15,83,89].

### 6.5. Summary of Human Evidence

The available evidence remains inconclusive. Findings vary according to the toxic element studied, the biological matrix analyzed and the study design. The strongest evidence concerns cadmium, for which positive associations with endometriosis have been reported in both population-based and biomonitoring studies. Although fewer studies have evaluated lead, the available findings also suggest a potential association with the disease. In contrast, evidence for mercury and arsenic remains limited. When interpreting these findings, it is important to recognize that individual studies rarely provide definitive answers. A more consistent picture emerges when epidemiological evidence is considered together with biomonitoring and experimental data. Although current evidence is insufficient to identify toxic elements as established risk factors for endometriosis, it supports the biological plausibility of their involvement in disease pathogenesis.

## 7. Discussion

### 7.1. What Does the Current Evidence Tell Us?

Endometriosis is a complex disease resulting from interactions among genetic, hormonal, immunological and environmental factors. In recent years, increasing attention has been paid to the potential contribution of chronic exposure to environmental contaminants, including toxic elements. Although the available evidence does not establish a causal relationship, it suggests that toxic elements may represent one of the environmental factors involved in the development or progression of endometriosis [62,90,91].

Among the toxic elements discussed, cadmium has been investigated most extensively. Recent epidemiological and biomonitoring studies generally support a possible association between cadmium exposure and endometriosis, although earlier studies have produced inconsistent findings. The available evidence for lead is less extensive but also suggests a possible association with the disease. In contrast, data on mercury and arsenic remain limited and are insufficient to draw firm conclusions (Table 2). Despite the heterogeneity of epidemiological studies in terms of design, exposure assessment, and study populations, experimental evidence supports several biological mechanisms through which toxic elements may contribute to the development and progression of endometriosis. Toxic elements have been shown to influence biological processes implicated in endometriosis, including endocrine disruption, chronic inflammation, oxidative stress, and epigenetic regulation [15,61,74].

Furthermore, biomonitoring studies have demonstrated the presence of toxic elements not only in blood and urine but also in follicular fluid and reproductive tissues. Taken together, these findings support the biological plausibility of an association between chronic exposure to toxic elements and endometriosis. However, the available evidence does not support a causal relationship, and toxic elements should not be regarded as independent etiological factors. Rather, they are likely to act together with genetic, hormonal, immunological, and other environmental factors that contribute to disease development and progression.

### 7.2. Why Are the Findings Inconsistent?

The inconsistency of the available evidence does not necessarily indicate the absence of an association between toxic elements and endometriosis. Rather, it is more likely to reflect methodological differences among studies as well as the complex nature of the disease itself [92]. One of the main reasons for this heterogeneity is the assessment of exposure. Different studies have used a variety of biomarkers, including blood, urine, follicular fluid, and reproductive tissues [93,94]. Since these biological matrices reflect different exposure windows, direct comparisons between studies are challenging. Blood concentrations generally indicate recent or short-term exposure, whereas urinary concentrations may better reflect longer-term exposure for some elements, particularly cadmium.

Most epidemiological studies measured total mercury or total arsenic rather than individual chemical species. However, the toxicity and biological behavior of these elements depend on their chemical form. In addition, total urinary arsenic may largely reflect relatively non-toxic organic arsenic derived from seafood. This may complicate the interpretation and comparison of biomonitoring results across studies. Measurements in follicular fluid and reproductive tissues provide information on local exposure within the female reproductive tract, but studies using these matrices have so far been limited by relatively small sample sizes [95].

Another important source of variability is the diagnosis of endometriosis. Some studies relied on self-reported diagnoses, whereas others confirmed endometriosis by laparoscopy or histopathological examination [92]. These differences increase the risk of disease misclassification and complicate comparisons across studies. Study populations also varied considerably, ranging from women in the general population to infertile patients, women undergoing IVF procedures, and occupationally exposed workers. In addition, participants differed in age, lifestyle, dietary habits, smoking status, iron status and the presence of comorbidities, all of which may have influenced both toxic element exposure and the risk of endometriosis (Table 2, Figure 1).

Most studies evaluated individual toxic elements, whereas real-life exposure involves complex mixtures of metals and other environmental contaminants that may exert additive or synergistic effects [5,58,62]. Moreover, few studies have considered factors influencing metal bioavailability, particularly iron status, despite evidence that iron deficiency may substantially increase cadmium absorption and accumulation [3,15,96].

Finally, most available studies were cross-sectional or case–control in design, precluding assessment of the temporal relationship between exposure and disease onset. Consequently, the available evidence remains insufficient to establish causality. In addition, residual confounding cannot be excluded, as factors such as smoking, dietary habits, iron status, renal function, and fertility treatment may have influenced the observed associations between toxic element exposure and endometriosis.

Despite these limitations, epidemiological studies, biomonitoring data, and experimental evidence collectively support the biological plausibility of a role for toxic elements in endometriosis. However, well-designed prospective studies with standardized exposure assessment are still needed to clarify the nature and strength of these associations.

### 7.3. Nutritional Perspective: Diet as a Modifier of Toxic Element Exposure

Diet plays a dual role in toxic element exposure. It is not only the primary source of exposure but also influences the absorption and retention of these elements in the body. Unlike many other environmental factors, dietary habits are modifiable, making nutrition an important target for public health strategies aimed at reducing toxic element exposure [97,98,99].

Chronic exposure to cadmium, lead, mercury and arsenic occurs primarily through the daily consumption of food and drinking water. Although the major dietary sources differ among individual elements, exposure is typically long-term and involves low doses accumulated over many years. This pattern of chronic, low-dose exposure is difficult to capture in epidemiological studies but may contribute to the development of chronic diseases, including endometriosis [5,15,24].

However, the internal body burden of toxic elements depends not only on the amount ingested but also on nutritional status. The best-characterized example is the relationship between iron homeostasis and cadmium absorption. Iron deficiency increases the expression of divalent metal transporter 1 (DMT1), thereby enhancing intestinal cadmium absorption [100]. This mechanism may be particularly relevant in women with endometriosis, in whom heavy menstrual bleeding and chronic blood loss frequently result in depleted iron stores [17,101]. These observations indicate that the assessment of toxic element exposure should consider nutritional status in addition to metal concentrations measured in biological samples. Consequently, similar levels of environmental exposure may not result in the same internal cadmium burden [54,102,103]. Differences in iron status may influence cadmium absorption, retention and accumulation, thereby modifying individual susceptibility to its biological effects [17,104].

Therefore, nutritional status should be taken into account when interpreting biomonitoring results in women with endometriosis. Measuring metal concentrations alone may not fully reflect internal exposure, particularly in women with depleted iron stores. Further studies are needed to determine whether this approach improves exposure assessment and risk evaluation.

### 7.4. Future Research Directions

Despite the growing body of evidence, the role of toxic elements in the pathogenesis of endometriosis remains incompletely understood. Future studies should include prospective cohorts with standardized biomonitoring methods and consistent diagnostic criteria to improve the comparability of findings [92].

Repeated exposure measurements and longer follow-up periods would also help clarify the temporal relationship between toxic element exposure and disease onset.

Future research should also move beyond the assessment of individual toxic elements and consider exposure to mixtures of environmental contaminants, which more closely reflects real-life conditions. Factors affecting metal bioavailability, particularly iron status, should also be taken into account, as they may substantially influence cadmium absorption and internal body burden [100].

Finally, combining biomonitoring with nutritional assessment may improve the interpretation of exposure data and help explain differences in individual susceptibility to toxic elements [62]. In the future, biomonitoring may complement emerging non-invasive diagnostic strategies by providing information on environmental exposure alongside molecular and clinical biomarkers. However, its diagnostic value in endometriosis remains to be established [105].

## 8. Conclusions

Chronic exposure to toxic elements may represent one of the environmental factors involved in the development and progression of endometriosis. Although the available evidence does not establish a causal relationship, findings from epidemiological, biomonitoring and experimental studies collectively support the biological plausibility of this association. Among the toxic elements reviewed, the evidence is strongest for cadmium, whereas data on lead, mercury, and arsenic remain limited.

This review highlights that the assessment of toxic element exposure should not be based solely on metal concentrations measured in biological samples. Nutritional status, particularly iron homeostasis, may influence cadmium absorption and internal body burden, thereby modifying individual susceptibility to environmental exposure. Considering nutritional status alongside biomonitoring data may improve the interpretation of exposure assessment and contribute to a better understanding of the role of toxic elements in endometriosis.

Future large prospective multicenter studies incorporating standardized biomonitoring protocols and nutritional assessment are needed to better define the contribution of toxic elements to the pathogenesis of endometriosis.

## Figures and Tables

**Table 1:** Characteristics of major toxic elements relevant to endometriosis, including dietary and environmental exposure sources, toxicokinetic properties, nutritional modifiers, and proposed biological mechanisms [2,3,15,21,23,24,25,26,27,28,29,30,31,32,33,34,35,36,37].

Characteristic	Cadmium (Cd)	Lead (Pb)	Mercury (Hg)	Arsenic (As)
main dietary contributors to chronic exposure	cereals (especially rice), leafy vegetables, seafood, organ meats	cereals (especially rice), vegetables, seafood, beverages	fish and seafood (especially predatory fish)	rice and rice products, seafood, drinking water
major environmental sources	mining, smelting, industrial manufacturing, agricultural practices, tobacco smoke,	mining, industrial manufacturing, natural sources, water pollution	coal combustion artisanal and small-scale gold mining, industrial emissions	geological sources (volcanic activity), stormwater runoff, agricultural practices
major target organs/ accumulation sites	kidneys, liver, lungs, bones	bones, teeth, blood, liver, kidney	brain, kidneys, reproductive tissues, placenta, spleen	skin, hair, brain, liver, kidneys, cardiovascular system
biological half-life (major compartment)	10–30 years	blood ~30 days; soft tissues: months, bone 20–30 years	methylmercury ~50 days (range: 30–120 days); inorganic mercury: years to decades	a few days (continuous exposure maintains body burden)
nutritional factors modifying absorption or toxicity	iron deficiency (↑DMT1), low zinc intake, low calcium intake, low selenium intake, low magnesium intake, low vit. E, B1 and D intake, low dietary fiber intake, low phytates intake, low polyphenols intake	low calcium intake, low iron intake, low zinc intake, low vitamin C intake, low dietary fiber intake	low selenium intake, low sulfur-containing nutrients intake, low curcumin intake, low omega-3 fatty acids intake	low iron intake, low selenium intake, low aluminum intake, low tannic acid intake, low B-vitamins intake, low one-carbon metabolism intake
main mechanisms implicated in endometriosis	endocrine disruption; oxidative stress; inflammation; impaired angiogenesis; epigenetic alterations; ferroptosis	oxidative stress; endocrine disruption; epigenetic alterations; immune dysregulation; mitochondrial dysfunction	oxidative stress; endocrine disruption; immune dysregulation; mitochondrial dysfunction; epigenetic alterations	oxidative stress; chronic inflammation; endocrine disruption; epigenetic alterations; impaired cellular signaling
human epidemiological evidence	most consistent association	inconsistent findings	limited human evidence	very limited human evidence

## Data Availability

No new data were created or analyzed in this study. Data sharing is not applicable to this article.

## Abbreviations

The following abbreviations are used in this manuscript:

CAT	catalase
DMT1	divalent metal transporter 1
GPx	glutathione peroxidase
IL	interleukin
ROS	reactive oxygen species
SOD	superoxide dismutase
